# Inhibition of the Primary Bile Acid Synthesis Pathways in SD Rats at Different Altitudes

**DOI:** 10.3390/ani16081167

**Published:** 2026-04-10

**Authors:** Piao Ma, Qingfei Hu, Fan Ma, Wenjuan Zhang, Haifeng Gu, Dengbang Wei, Zhifang An

**Affiliations:** 1 State Key Laboratory of Plateau Ecology and Agriculture, Qinghai University, Xining 810016, China; 2 Qinghai Provincial Key Laboratory of Animal Ecological Genomics, Northwest Institute of Plateau Biology, Chinese Academy of Sciences, Xining 810008, China

**Keywords:** Sprague-Dawley rats, serum, bile acids, bile acid synthesis pathway

## Abstract

Bile acids, the primary constituents of mammalian bile, are synthesized in the liver from cholesterol and secreted into the intestine to perform essential physiological functions. Primary bile acid synthesis is the principal pathway for cholesterol catabolism and whole-body cholesterol homeostasis, occurring predominantly via the classical and alternative pathways. To elucidate the effects of altitude on serum bile acid profiles and synthesis pathways in SD rats, this study utilized UPLC-MS/MS to analyze serum bile acid composition in animals housed at high and low altitudes. Additionally, qRT-PCR and Western blotting assessed mRNA transcription and protein expression of key genes involved in primary bile acid synthesis in the liver and intestinal tissues (ileum, duodenum, and colon). Results showed that serum levels of total and primary bile acids significantly decreased with increasing altitude. Furthermore, hepatic mRNA and protein expression of *Cyp7a1*, *Cyp8b1*, *Cyp27a1*, and *Cyp7b1* were significantly downregulated. *Fxr* mRNA expression in the liver, ileum, duodenum, and colon was significantly decreased with increasing altitude. Meanwhile, the protein expression of both FGF15 and SHP showed a downward trend, with a significant decrease for FGF15 and a non-significant decrease for SHP. These findings suggest that primary bile acid synthesis in SD rats is dominated by the classical pathway. As altitude increases, bile acid synthesis in SD rats is significantly inhibited, indicating that high-altitude hypobaric hypoxia is the primary inhibitory factor. This study provides critical data for elucidating the adaptive mechanisms of bile acid metabolism in mammals exposed to high-altitude hypoxia, thereby establishing a theoretical foundation for investigating the regulation of host lipid metabolism influenced by such conditions.

## Simple Summary

Bile acid metabolism plays an essential role in maintaining energy homeostasis, especially under the hypoxic stress characteristic of high-altitude environments. This study utilized Sprague-Dawley (SD) rats to investigate the effects of high-altitude exposure on primary bile acid metabolism. Analysis of serum bile acid composition and levels, along with the evaluation of key gene and protein expression in hepatic and intestinal tissues, demonstrated that high-altitude exposure significantly decreased both total and primary bile acid levels. Concurrently, key bile acid synthesis genes (e.g., *Cyp7a1*, *Cyp8b1*) and proteins (e.g., FXR, FGF15) were significantly downregulated, leading to an altered CA/CDCA ratio. These findings indicate that high-altitude hypoxia inhibits the classical bile acid synthesis pathway and modifies bile acid profiles. This remodeling of the bile acid profile may disrupt lipid metabolic homeostasis.

## 1. Introduction

Bile acids (BAs) are steroidal compounds derived from cholesterol metabolism in the liver and gut microbiota, predominantly existing as sodium or potassium salts [1,2]. Research indicates that environmental factors modulate the content and composition of bile acids in both humans and animals [1,2], either directly or indirectly through alterations in gut microbiota. The profiles and abundance of bile acids vary among species and correlate with environmental adaptations. The liver and intestine serve as the primary sites for bile acid metabolism; primary bile acids are synthesized in the liver, transported to the intestine via enterohepatic circulation, and converted into secondary bile acids by gut microbiota. Hepatic primary bile acid synthesis occurs through two distinct pathways, the classic (neutral) and alternative (acidic) pathways, which involve at least 17 enzymes [2,3,4]. The classical pathway begins with the conversion of cholesterol to 7α-hydroxycholesterol by cholesterol 7α-hydroxylase (CYP7A1), which subsequently yields cholic acid (CA) and chenodeoxycholic acid (CDCA). As the rate-limiting enzyme, CYP7A1 regulates total bile acid biosynthesis; under normal physiological conditions, this pathway contributes to at least 75% of bile acid production [2,3,4,5]. Conversely, the alternative pathway is initiated by sterol 27-hydroxylase (CYP27A1), which produces 27-hydroxycholesterol. This compound is further metabolized by oxysterol 7α-hydroxylase (CYP7B1) to form CDCA. The ratio of CA to CDCA is contingent upon sterol 12α-hydroxylase (CYP8B1), an enzyme that is essential for the synthesis of CA. Although CYP7A1 serves as a rate-limiting enzyme and is regulated by the gut microbiota, CYP8B1 remains independent of microbial regulation [2,3,4,6,7].

Bile acid synthesis is subject to negative feedback regulation by the nuclear receptor Farnesoid X receptor (FXR), a transcription factor that binds to promoter regions to regulate target gene expression. FXR is predominantly expressed in the liver, kidney, and ileum, while exhibiting lower expression levels in other tissues [2,3,8]. FXR modulates bile acid metabolism through two distinct mechanisms. First, the activation of hepatic FXR by bile acids induces the expression of Small Heterodimer Partner (SHP), which subsequently binds to Liver Receptor Homolog-1 (LRH-1) to inhibit the transcription of cytochrome P450 family 7 subfamily A member 1 (CYP7A1). Second, FXR activation in the distal ileum induces the expression of fibroblast growth factor 15 or 19 (FGF15/19; FGF15 in rodents and FGF19 in humans). FGF15/19 travels to the liver via the portal vein and binds to the fibroblast growth factor receptor 4 (FGFR4)/β-Klotho heterodimer complex, triggering c-Jun N-terminal kinase 1/2 (JNK1/2) and extracellular signal-regulated kinase 1/2 (ERK1/2) signaling cascades that repress *Cyp7a1* expression. Studies utilizing tissue-specific *Fxr*-deficient mice have demonstrated that ileal FXR activation inhibits CYP7A1-mediated bile acid synthesis more potently than hepatic FXR activation, whereas hepatic FXR likely modulates cytochrome P450 family 8 subfamily B member 1 (CYP8B1) to alter CA synthesis. CDCA is the most potent endogenous FXR agonist, followed by CA, deoxycholic acid (DCA), and lithocholic acid (LCA), while ursodeoxycholic acid (UDCA) inhibits *FXR* activation. The taurine-conjugated primary bile acids, tauro-α-muricholic acid (Tα-MCA) and tauro-β-muricholic acid (Tβ-MCA), act as natural FXR antagonists [2,4,9,10]. Furthermore, FXR activation induces the expression of additional bile acid transporters, thereby modulating bile acid metabolism. The interaction between the microbiota and bile acids is bidirectional; bile acids reshape the gut microbial community by promoting the growth of bile acid-metabolizing bacteria while inhibiting the proliferation of bile-sensitive taxa [2,4,9,10]. In conclusion, the composition and abundance of the gut microbiota dictate the bile acid profile and content. Specifically, microbial enzymes catalyze the biotransformation of primary bile acids synthesized in the liver into a broad spectrum of secondary bile acids.

Our previous studies indicated that primary bile acid synthesis in high-altitude animals (plateau zokors and plateau pikas) occur predominantly via the alternative pathway. Furthermore, inhibition of the classical bile acid synthesis pathway intensifies significantly with increasing altitude [11]. Significant interspecies differences in serum secondary bile acid concentrations were observed between these two species, with levels declining significantly as altitude increased; this suggests variations in gut microbiota composition both between species and across habitats [11]. Using SD rats as a model, this study examined the effects of high-altitude hypoxia on primary bile acid metabolism by analyzing serum bile acid profiles as well as mRNA and protein expression of key synthetic enzymes across different altitudinal gradients. These findings provide an experimental basis for understanding altitude-related bile acid metabolism and for safeguarding the health of high-altitude populations.

## 2. Materials and Methods

### 2.1. Laboratory Animals

Male SD rats (mean body weight, 160 ± 3 g) were procured from Beijing HFK Bio-Technology Co., Ltd. (Beijing, China). The rats were randomly assigned to two groups: a low-altitude group (RL) and a high-altitude group (RH). The RL group was housed in an animal facility at Qinghai University, located in Xining, Qinghai Province (~2200 m; 36°62′ N, 101°77′ E; atmospheric pressure, 75 kPa; oxygen content, ~196.5 g/m^3^), whereas the RH group was housed at Laji Mountain in Guide County, Hainan Prefecture, Qinghai Province (~3700 m; 36°21′ N, 101°26′ E; atmospheric pressure, 59 kPa; oxygen content, ~173.1 g/m^3^). Each group consisted of 6 rats (*n* = 6), and the animals were maintained indoors for a duration of 20 days [12,13].

Following intraperitoneal anesthesia with 20% urethane, serum and tissue samples were harvested from all experimental animals and immediately snap-frozen in liquid nitrogen. All procedures involved in the handing and care of animals were in accordance with the China Practice for the Care and Use of Laboratory Animals and approved by the China Zoological Society (permit number: GB/T 35892-2018 [14].The disposal of animal carcasses was recorded and managed by the Animal Experiment Management Office of the State Key Laboratory at Qinghai University.

### 2.2. Determination of Bile Acid Content

(1) Sample Preparation: Serum samples were thawed on ice, and 50 μL aliquots were transferred into microcentrifuge tubes. Subsequently, 300 μL of ice-cold methanol was added to each tube, and the mixtures were vortexed for 1 min and sonicated at 4 °C for 30 min. Following centrifugation at 12,000 rpm for 10 min, the supernatant was collected and evaporated to dryness. The residues were reconstituted in 200 μL of methanol containing an internal standard at a concentration of 50 ng/mL (Merck, Darmstadt, Germany). Following reconstitution, the samples were centrifuged for 15 min, and the resulting supernatant was analyzed using UPLC-MS/MS (Vanquish Core UHPLC-TSQ Quantis, Thermo Fisher Scientific, Waltham, MA, USA).

(2) Chromatographic Conditions: Samples were maintained at 4 °C in an autosampler. Separation was achieved using a Waters Acquity UPLC column under the following parameters: injection volume of 4 μL, column temperature set at 40 °C, and a flow rate of 0.3 mL/min. The mobile phases consisted of 0.1% formic acid in water (solvent A) and 0.1% formic acid in methanol (solvent B). The gradient elution program was implemented as follows: 0–1 min, 10% B; 1–2 min, 40% B; 2–5 min, 45% B; 5–7.5 min, 60% B; 7.5–9.5 min, 65% B; 9.5–11.5 min, 80% B; 11.5–13.5 min, 80% B; 13.5–14 min, 10% B; and 14–16 min, 10% B.

(3) Mass Spectrometry Conditions: Detection was performed using electrospray ionization (ESI) in multiple reaction monitoring (MRM) mode. The ion source parameters were set as follows: curtain gas, 35 arb; collision gas, 7 arb; ion spray voltage, −4200 V; ion source temperature, 450 °C; ion source gas 1 (GS1), 35 arb; and ion source gas 2 (GS2), 35 arb.

Using the established chromatographic and mass spectrometry conditions, standard solutions were injected to determine the retention times of the analytes. Peak integration was performed using MultiQuant 3.0.3 software, and quantification was achieved using the internal standard single-point method.

### 2.3. mRNA and Protein Expression Levels of Bile Acid Synthesis Enzyme Genes

#### 2.3.1. Design and Synthesis of Primers

Primers specific for *Cyp7a1* (NM_012942.2), cytochrome P450 family 27 subfamily A member 1 (*Cyp27a1*) (NM_178847.3), *Cyp7b1* (NM_178847.3), cytochrome P450 family 7 subfamily B member 1 (*Cyp8b1*) (NM_031241.2), *Fxr* (NM_021745.1), and *β-actin* (NM_031144.3) in SD rats were designed using Beacon Designer 8 software, based on sequences obtained from GenBank. The primer sequences (Table 1) were synthesized by Sangon Biotech (Shanghai) Co., Ltd. (Shanghai, China).

#### 2.3.2. Determination of mRNA Expression Levels

Total RNA was extracted from the tissues of SD rats using a Total RNA Extraction Kit (Tiangen, Beijing, China). RNA concentration (>0.4 μg/μL) and purity (A260/A280 ratio, 1.8–2.0) were assessed using a nucleic acid/protein analyzer (NanoPhotometer^®^ NP80, Implen, München, Germany). First-strand cDNA was synthesized from 4 μg of total RNA using the FastKing One-Step gDNA Removal and cDNA Synthesis SuperMix (Tiangen, Beijing, China). PCR amplification was conducted using the Premix Ex Taq kit (Takara, Shiga, Japan) to prepare standards.

Amplified products were separated by gel electrophoresis and purified using a Common Agarose Gel DNA Recovery Kit (Tiangen, Beijing, China). The undiluted product was assigned a relative concentration of 1 and subjected to 10-fold serial dilutions to generate 10 standards ranging from 1 × 10^−1^ to 1 × 10^−10^. Quantitative real-time PCR (qPCR) was conducted on an iQ5 Multicolor Real-Time PCR Detection System (iQ5, Bio-Rad, Hercules, CA, USA) utilizing the TB Green^®^ Premix Ex Taq™ II (Tli RNaseH Plus) kit (Takara, Shiga, Japan), in accordance with the manufacturer’s instructions. The 25 μL reaction mixture contained 12.5 μL of SYBR Premix Ex Taq II, 0.5 μL of each primer, and 1 μL of cDNA, with the volume adjusted using ddH_2_O. The thermal cycling conditions were as follows: initial denaturation at 95 °C for 3 min, followed by 40 cycles of 95 °C for 30 s, 60 °C for 30 s, and 72 °C for 30 s [15]. Data acquisition and analysis were performed using Bio-Rad CFX Manager 2.1 software to quantify the concentrations of target and housekeeping genes. Relative expression levels were calculated by normalizing the concentrations of target genes to those of the housekeeping gene.

#### 2.3.3. Determination of Protein Expression Levels

Total protein was extracted using a Total Protein Extraction Kit (KeyGen, Nanjing, China) and quantified with the Pierce™ BCA Protein Assay Kit (Thermo Fisher Scientific, Waltham, MA, USA). Proteins (20–50 µg) were separated by SDS-polyacrylamide gel electrophoresis (SDS-PAGE) (041BR147752, Bio-Rad, Hercules, CA USA) and subsequently transferred to 0.45 µm PVDF membranes. The membranes were blocked with 5% skim milk at 37 °C for 3 h with gentle agitation. Subsequently, membranes were incubated with primary antibodies overnight at 4 °C: CYP7A1 (orb539102, Biorbyt, Cambridge, UK), CYP27A1 (orb539093, Biorbyt), CYP7B1 (orb542525, Biorbyt), CYP8B1 (orb213831, Biorbyt), FXR (orb156973, Biorbyt), FGF15 (ab229630, Abcam, Cambridge, UK), SHP (DF6648, Affinity, San Francisco, CA, USA), and β-actin (ab8227, Abcam). After washing with TBST, membranes were incubated with secondary antibodies (Servicebio, Wuhan, China) diluted in 1 × TBST at room temperature for 2 h, followed by a final wash. Protein bands were visualized using an ECL Fluorescence Kit (Boster, Wuhan, China) and detected with a FluorChem R system (FluorChem R, ProteinSimple, San Jose, CA, USA). The membranes were exposed in triplicate, and the signal intensity was averaged [16]. Finally, relative protein expression levels were quantified by measuring grayscale values using ImageJ 1.54s software.

### 2.4. Statistical Analysis

Data were processed using Excel 2021 and analyzed using SPSS 25. Data normality and homogeneity of variance were assessed using the Kolmogorov–Smirnov and Levene’s test, respectively. Differences between altitudes were evaluated using an independent-samples *t*-test. Data are presented as mean ± standard deviation (SD). Statistical significance was set at *p* < 0.05.

## 3. Results

### 3.1. Primary Bile Acid Profile in the Serum of SD Rats

A total of eleven primary bile acids were identified in the serum of SD rats from both the high-altitude hypoxia exposure group and the low-altitude group. These bile acids include CA, TCA, GCA, CDCA, TCDCA, GCDCA, UDCA, βUDCA, αMCA, βMCA, and Tα(β)MCA (Table 2). Notably, CA was present at the highest concentration in rat serum.

### 3.2. Contents of Total Bile Acids, Primary Bile Acids, CA Class, CDCA Class, UDCA Class, and MCA in SD Rat Serum

**Figure 1:**
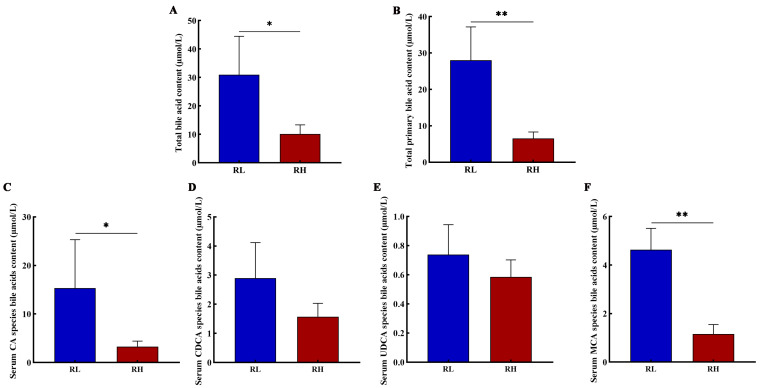
The total bile acid contents (**A**), the primary bile acids contents (**B**), cholic acid (CA) (**C**), chenodeoxycholic acid (CDCA) (**D**), ursodeoxycholic acid (UDCA) (**E**), and muricholic acid (MCA) (**F**) in the serum of SD rat at different altitudes. RL and RH denote SD rats from the low-altitude and high-altitude groups, respectively, *n* = 6. Differences between the groups were analyzed using an independent-samples *t*-test, with significance levels indicated as * *p* < 0.05, ** *p* < 0.01.

The results indicated that the concentrations of total bile acids and total primary bile acids in the serum of SD rats from the high-altitude group were significantly lower than from the low-altitude group. As altitude increased, the serum concentrations of primary bile acids, including CA and MCA, were significantly decreased in SD rats (Figure 1).

### 3.3. Contents and Ratio Variations of CA and CDCA Classes, Conjugated and Unconjugated Bile Acids, 12-Hydroxy and Non-12-Hydroxy Bile Acids, and G-BAs to T-BAs in SD Rat Serum

**Figure 2:**
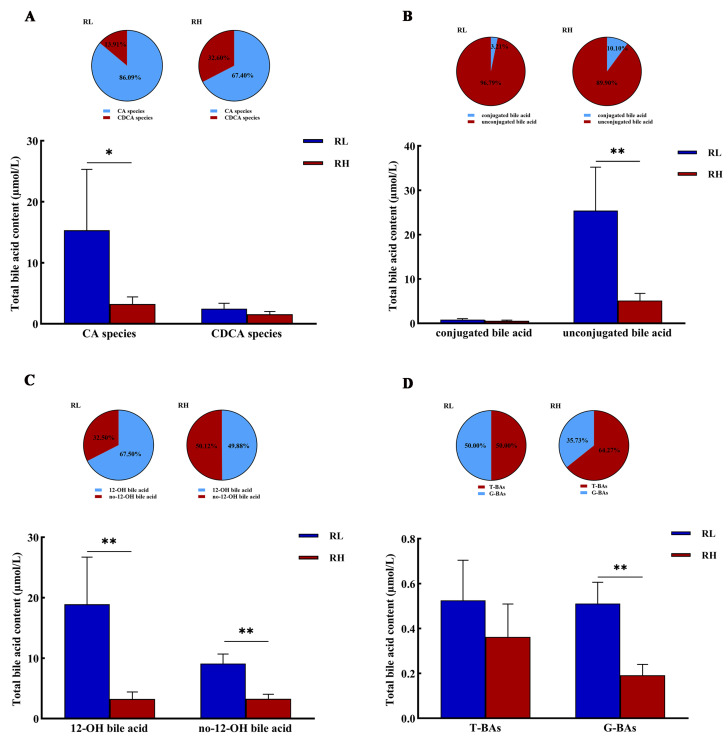
The ratio and contents of cholic acid (CA) and chenodeoxycholic acid (CDCA) species bile acids (**A**), conjugated bile acids and unconjugated bile acids (**B**), 12-OH bile acids and no-12-OH bile (**C**), and glycine conjugated bile acid (G-BAs) and taurine conjugated bile acid (T-BAs) (**D**) in the serum of SD rats at different altitudes. RL and RH denote SD rats from the low-altitude and high-altitude groups, respectively, *n* = 6. Differences between the groups were analyzed using an independent-samples *t*-test, with significance levels indicated as * *p* < 0.05, ** *p* < 0.01.

In the serum of SD rats, the ratio of CA to CDCA was observed to exceed 6 in the low-altitude group but decreased to 2 in the high-altitude group. This finding indicates that the proportion of CDCA increased while that of CA decreased with increasing altitude (Figure 2A). As altitude increased, the proportion of conjugated bile acids rose, whereas that of unconjugated bile acids declined; however, the concentrations of unconjugated bile acids remained significantly higher than those of conjugated bile acids (Figure 2B). Furthermore, the proportion of 12-hydroxy bile acids decreased, while that of non-12-hydroxy bile acids increased with increasing altitude (Figure 2C). Although taurine-conjugated bile acids (T-BAs) and glycine-conjugated bile acids (G-BAs) each accounted for approximately 50% of the total in the low-altitude group, the T-BA-to-G-BA ratio exceeded 1.5 in the high-altitude group. This change was accompanied by a significant increase in T-BA concentrations and a significant decrease in G-BA concentrations as altitude rose (Figure 2D).

### 3.4. mRNA and Protein Expression Levels of Bile Acid Synthesis Enzyme Genes in Rat Liver Tissue

**Figure 3:**
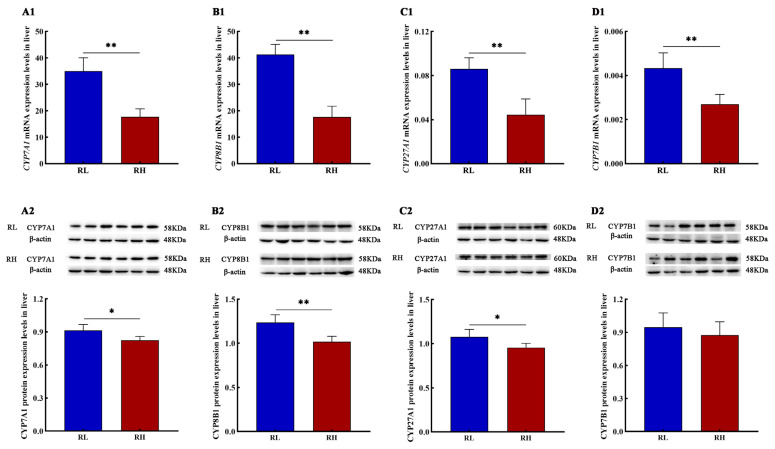
The mRNA and proteins expression levels of bile acid synthesis genes in the liver of SD rats. (**A1**), (**B1**), (**C1**) and (**D1**) represent the mRNA expression levels of cytochrome P450 family 7 subfamily A member 1 (*Cyp7a1*), cytochrome P450 family 8 subfamily B member 1 (*Cyp8b1*), cytochrome P450 family 27 subfamily A member 1 (*Cyp27a1*) and cytochrome P450 family 7 subfamily B member 1 (*Cyp7b1*) in the liver of SD rats; (**A2**), (**B2**), (**C2**) and (**D2**) represent the protein expression levels of CYP7A1, CYP8B1, CYP27A1 and CYP7B1 in the liver of SD rats, respectively (*n* = 6). RL and RH denote SD rats from the low-altitude and high-altitude groups, respectively, *n* = 6. Differences between the groups were analyzed using an independent-samples *t*-test, with significance levels indicated as * *p* < 0.05, ** *p* < 0.01.

The results demonstrated that the mRNA expression levels of *Cyp7a1*, *Cyp8b1*, *Cyp27a1*, and *Cyp7b1* in SD rat liver tissues significantly decreased with increasing altitude. Similarly, protein expression levels of CYP7A1, CYP8B1, and CYP27A1 were significantly reduced (Figure 3).

### 3.5. Relative Expression Levels of FXR Gene and Protein in Different Tissues of SD Rats

**Figure 4:**
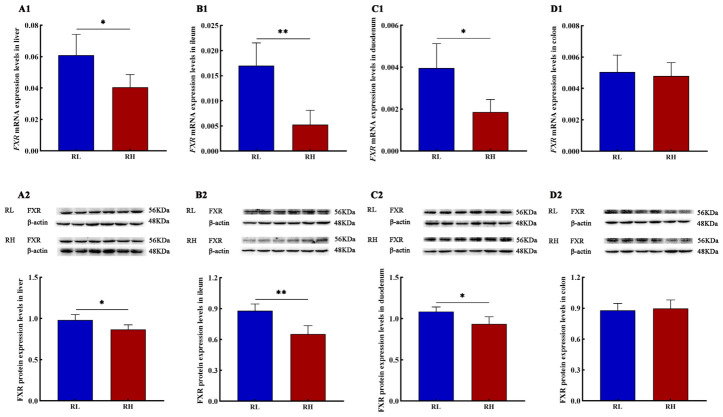
The mRNA and protein expression levels of farnesoid X receptor (*Fxr*) in the different tissues of SD rats. (**A1**), (**B1**), (**C1**) and (**D1**) represent the mRNA expression levels of *Fxr* in the liver, ileum, duodenum and colon of SD rats; (**A2**), (**B2**), (**C2**) and (**D2**) represent the protein expression levels of FXR in the liver, ileum, duodenum and colon of SD rats, respectively, *n* = 6. RL and RH denote SD rats from the low-altitude and high-altitude groups, respectively. Differences between the groups were analyzed using an independent-samples *t*-test, with significance levels indicated as * *p* < 0.05, ** *p* < 0.01.

The results indicated that FXR expression was most abundant in the livers of SD rats. mRNA levels of *Fxr* significantly decreased with increasing altitude across all examined tissues, including the liver, ileum, duodenum, and colon. At the protein level, FXR expression was suppressed with increasing altitude in the liver, ileum, and duodenum (Figure 4).

### 3.6. Relative Protein Expression Levels of FGF15 and SHP in the Liver Tissues of SD Rats

**Figure 5:**
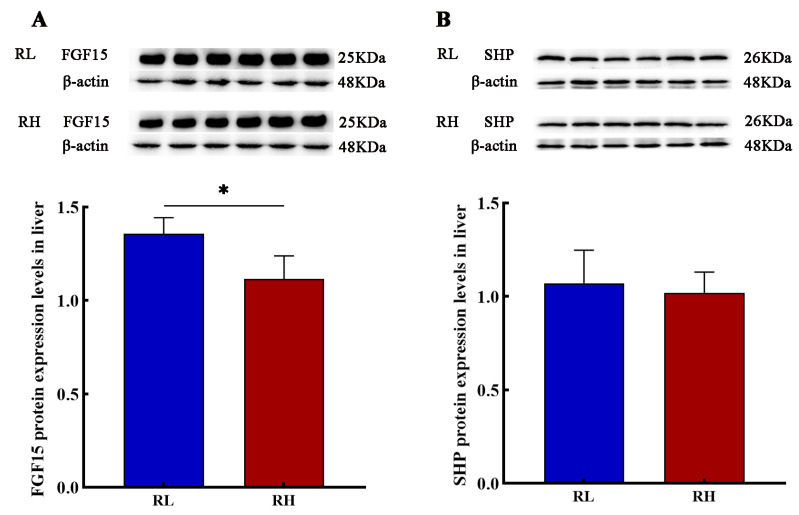
The protein expression levels of fibroblast growth factor 15 (FGF15) and small heterodimer partner (SHP) in the liver of SD rats. (**A**) and (**B**) represent the protein expression levels of FGF15 and SHP in the liver of SD rats, respectively (*n* = 6). RL and RH denote SD rats from the low-altitude and high-altitude groups, respectively. Differences between the groups were analyzed using an independent-samples *t*-test, with significance levels indicated as * *p* < 0.05.

Hepatic FGF15 protein expression in SD rats was significantly decreased with increasing altitude, whereas SHP protein expression in the high-altitude group showed a non-significant downward trend (Figure 5).

## 4. Discussion

Bile acids, a class of sterol compounds, are synthesized from cholesterol through hepatic metabolism and subsequent microbial transformation in the gut. In addition to their role in facilitating the absorption of fat-soluble nutrients, bile acids function as essential signaling molecules that regulate various physiological processes, including endogenous bile acid metabolism, glucose and lipid homeostasis, immune responses, and energy balance [17,18,19,20,21,22,23]. Environmental factors can modulate bile acid content and composition in both humans and animals, either directly or indirectly, with the latter mediated through alterations in gut microbiota composition [1,2]. The high-altitude environment of the Qinghai–Tibet Plateau significantly impacts animal physiology, evolution, and adaptation, inducing physiological and genetic modifications in response to environmental stressors such as hypoxia and cold exposure. High-altitude hypoxia has been shown to alter gut microbiota diversity and function in humans [24] and various mammalian species, including Tibetan antelope, wild ass, macaque, and yak [13,25,26,27], while acute exposure to high altitude additionally affects bile acid composition [28]. Bile acids serve as essential mediators of the gut–liver axis, regulating hepatic metabolism and influencing the composition of gut microbiota. Conversely, the gut microbiota modulates hepatic function by altering bile acid profiles and regulating immune responses [28]. This bidirectional interaction between bile acids and the gut microbiome constitutes a fundamental mechanism for maintaining digestive function and metabolic homeostasis.

Hepatic synthesis of primary bile acids occurs through both classical and alternative pathways. CYP7A1 serves as the rate-limiting enzyme in the classical pathway, thereby determining the overall production of bile acids [2,3,4]. The alternative pathway primarily involves two key enzymes: CYP27A1 and CYP7B1. This pathway predominantly generates CDCA, while the classical pathway produces both CDCA and CA. Additionally, CYP8B1 plays a crucial role in regulating the ratio of CA to CDCA. Inhibition of CYP8B1 can shift bile acid synthesis towards the alternative pathway, resulting in increased bile acid production via this route [2,3,4,6,7]. In the present study, serum concentrations of total bile acids and primary bile acids in SD rats were found to significantly decrease with increasing altitude. Measurement of mRNA expression levels of key enzymes involved in bile acid synthesis revealed that hepatic mRNA expression of *Cyp7a1*, *Cyp8b1*, *Cyp27a1*, and *Cyp7b1* was markedly downregulated in SD rats. At the protein level, the expression of CYP7A1, CYP8B1, and CYP27A1 was significantly suppressed with rising altitude. In the liver tissue of SD rats, CYP7A1 and CYP8B1 were predominantly expressed, while the expression level of CYP7B1 remained extremely low. Inhibition of CYP7A1 expression reduces bile acid production. As amphipathic molecules, bile acids facilitate the solubilization, digestion, and absorption of dietary lipids and fat-soluble vitamins. A reduction in bile acid levels impairs the emulsification efficiency of lipids in the intestine [29], thereby inhibiting lipid digestion and absorption. Inhibition of CYP8B1 promotes increased synthesis of CDCA via the alternative pathway. Given that CDCA exhibits lower emulsification activity than CA, this shift further compromises lipid digestion and absorption. Our results indicate that in SD rats, the serum CA-to-CDCA ratio among primary bile acids was approximately 6 in the low-altitude group. As altitude increased, the proportion of CA decreased while that of CDCA increased. Concurrently, significant downregulation of *Cyp7a1*, *Cyp8b1*, and *Cyp27a1* resulted in a reduced CA proportion and an elevated CDCA proportion, indicating suppression of the classical pathway. Our previous research demonstrated that primary bile acid synthesis in high-altitude animals, such as plateau zokors and plateau pikas, is primarily driven by the alternative pathway, with classical pathway inhibition intensifying at higher altitudes [11]. Collectively, these findings suggest that bile acid synthesis in SD rats predominantly relies on the classical pathway, which is suppressed as altitude increases. Consequently, the high-altitude environment may inhibit hepatic primary bile acid synthesis in SD rats, leading to reduced total levels and altered compositional ratios, thereby impairing intestinal lipid emulsification, digestion, and absorption.

In humans and other mammals, receptors such as FXR, TGR5, and VDR mediate the regulation of glucose and lipid metabolism, as well as energy homeostasis, through the binding of bile acids [2,20,29,30]. As a pivotal nuclear receptor that is highly expressed in the liver and intestine, FXR plays a crucial role in maintaining bile acid pool homeostasis by negatively regulating key enzymes such as CYP7A1 [2,3,8]. Our results revealed that serum levels of CDCA-type bile acids in SD rats varied significantly with altitude, while UDCA-type bile acids exhibited a decreasing trend that lacked statistical significance. Notably, both CDCA, the most potent FXR agonist, and UDCA, the most potent FXR antagonist, showed a decline in concentration with increasing altitude, leading to subsequent suppression of *Fxr* expression. In this study, we evaluated *Fxr* gene and protein levels in the liver, ileum, duodenum, and colon. The results indicated that *Fxr* mRNA expression in the liver, ileum, duodenum, and colon of SD rats significantly decreased with altitude, with the liver displaying the highest expression levels. At the protein level, the expression of FXR in the liver, ileum, and duodenum was concurrently inhibited by high altitude. These findings suggest that both classical and alternative bile acid metabolic pathways are suppressed in SD rats as altitude increases, potentially through FXR-dependent mechanisms. This downregulation of the FXR signaling pathway may be a critical factor contributing to the reduction in total hepatic bile acid synthesis and the alteration of CA/CDCA ratios.

Highly expressed in the liver and small intestine, SHP is a key regulator of metabolic homeostasis, involved in bile acid homeostasis, fatty acid and glucose metabolism, and xenobiotic detoxification [31,32]. Both FXR and SHP are intricately linked to bile acid metabolism, wherein SHP functions both as a transcriptional repressor and a direct target of FXR [33]. Our results demonstrate that the high-altitude hypoxic environment significantly inhibits the classical pathway of bile acid synthesis in SD rats, a mechanism primarily driven by the disruption of the FXR-mediated gut–liver signaling axis. High-altitude-induced hypoxia suppresses hepatic *Fxr* and its target gene, *Shp*, which is accompanied by a significant downregulation of intestinal FXR-regulated *Fgf15*. These findings are consistent with observations of inhibited FXR and SHP expression in HepG2 cell hypoxia models [34], indicating that hypoxia is a critical environmental regulator of this signaling axis. Interestingly, the SHP protein expression in this study exhibited only a declining trend without reaching statistical significance. This, along with evidence that SHP knockout mice do not exhibit significant cholestasis under basal conditions [33], suggests that the downregulation of the FXR-FGF15 signaling axis plays a dominant role in inhibiting the classical pathway in SD rats at high altitude, whereas the FXR/SHP axis functions synergistically. This further supports the notion that the FXR/SHP axis is not the sole regulator of bile acid metabolism.

With increasing altitude, serum bile acid synthesis is significantly inhibited, and high-altitude hypoxia likely represents a major inhibitory factor. Downregulated expression of genes related to the gut–liver FXR axis suppresses the classical pathway, resulting in reduced total bile acid synthesis and an altered BA composition profile. These alterations may impair intestinal lipid emulsification, digestion, and absorption.

## 5. Conclusions

In conclusion, the primary bile acid synthesis pathway in SD rats is dominated by the classical pathway. As altitude increases, bile acid synthesis in the serum of SD rats is significantly inhibited. This inhibition may be linked to the downregulation of key enzyme genes, such as *Cyp7a1* and *Cyp8b1*, alterations in bile acid composition, and the suppression of the FXR along with its downstream intestinal FXR-FGF15 signaling axis. These findings provide novel experimental evidence regarding the metabolic adaptation characteristics of animals in high-altitude environments.

## Figures and Tables

**Table 1:** Primer sequences of real-time PCR.

Species	Genes	Sense Primer (5′ to 3′)	Anti-Sense Primer (5′ to 3′)
SD rats	*C* *yp* *27* *a* *1*	GCCATCTGCTATATCCTGTT	TGACATAGACTGAGTTCTTGAA
	*C* *yp* *7* *b* *1*	TGACGACCTTAGCAATGA	CTGTATTCCAATCTGTGAGTT
	*C* *yp* *7* *a* *1*	TAATCCTCTTGAGTTCCTAA	TCGCAGAAGTAGTGTAAT
	*C* *yp* *8* *b* *1*	CTCTTCCACTTCTGCTACA	GCTCACTTCTACCCACTC
	*FXR*	CTCTCCAGACAGACAATACA	CGTGGTGATGGTTGAATG
	*β-actin*	TCACCAACTGGGACGATATG	GTTGGCCTTAGGGTTCAGAG

**Table 2:** Primary bile acid species are present in the serum of SD rats.

Name of Bile Acids	Abbreviation
Cholic acid	CA
Taurocholic acid	TCA
Glycocholic acid	GCA
Chenodeoxycholic acid	CDCA
Taurochenodeoxycholic acid	TCDCA
Glycochenodeoxycholic acid	GCDCA
Ursodeoxycholic acid	UDCA
β-Ursodeoxycholic acid	βUDCA
α-Muricholic acid	αMCA
β-Muricholic acid	βMCA
Tauro α(β)-muricholic acid	Tα(β)MCA

Underlined values indicate the primary bile acids with the highest serum concentrations.

## Data Availability

The data supporting the findings of this study are available within the article.
